# 2257. Cost-Benefit Analysis of Fidaxomicin (FDX) as First Line Treatment for Initial Episode of *Clostridioides difficile* Infection (CDI) during the COVID-19 Pandemic: A Quality Control/Improvement (QC/QI) Project at a Tertiary Care Veterans Affairs Medical Center (VAMC) over a 4-year period

**DOI:** 10.1093/ofid/ofad500.1879

**Published:** 2023-11-27

**Authors:** Sumaiya F Khaja, Ryan Kuhn, Sajjad Ali, Suganthini Krishnan Natesan

**Affiliations:** Wayne State University School of Medicine, Rochester Hills, Michigan; John D Dingell VA Medical Center, Detroit, Michigan; Wayne State University, Detroit, Michigan; John D Dingell VAMC/Wayne State University, Detroit, Michigan

## Abstract

**Background:**

In 2021, *Clostridioides difficile* infection treatment guidelines were updated to recommend fidaxomicin over oral vancomycin as first-line therapy for initial and recurrent episodes of CDI. The aim of this analysis is to evaluate the cost benefit advantage of using fidaxomicin as first line therapy for the first episode of CDI.

**Methods:**

A retrospective review of outpatient oral vancomycin and fidaxomicin use at the VAMC in Detroit, Michigan was conducted by evaluating total prescription fills per 1000 unique patients from 2019 to 2022. This study was done during the COVID-19 pandemic when there was an increased use of antibiotics for respiratory infections, and after the updated IDSA guidelines recommending fidaxomicin as first line therapy for initial and first recurrence of CDI. Diagnosis of CDI was based on clinical presentation and a positive stool C diff PCR test. Despite substantial cost of FDX, the expectation was that there would be a significant reduction in recurrent CDI, hospitalizations, length of stay, all of which would offer a cost-benefit advantage.

**Results:**

There was a 5-fold increase in the use of fidaxomicin in 2021-2022 compared to 2018-2019. Its use was directly proportional to the increased use of respiratory quinolones and Beta lactam antibiotics for respiratory infections during the COVID-19 pandemic in 2020. Insurance co-pays were high and the FDX company support programs failed to cover veterans with Medicaid/Medicare coverage. Veterans were unable to complete the 10-day course of FDX as it was unaffordable. They had to be transitioned to oral vancomycin to complete the 10-day course for CDI.

**Conclusion:**

Reduction of recurrent CDI may not offset the higher up-front cost of fidaxomicin ($2,400/10 days), compared to oral vancomycin ($30/10 days). This QC/QI analysis over a 4-year period revealed that increased use of fidaxomicin as first-line treatment for CDI did not translate into a cost advantage through decreases in CDI recurrence, given the high cost of fidaxomicin. Reduction in cost of fidaxomicin to < $1,200 may be needed to achieve the expected cost benefit. Based on our data, starting November 2022, FDX has been restricted in our institution. Prescription for FDX requires ID approval, and is reserved for recurrent CDI and not for the first episode of CDI.

**Disclosures:**

**All Authors**: No reported disclosures

